# Data justice and data solidarity

**DOI:** 10.1016/j.patter.2021.100427

**Published:** 2022-03-11

**Authors:** Matthias Braun, Patrik Hummel

**Affiliations:** 1 Research Group Ethics and Governance of Emerging Technologies, Department of Systematic Theology, Friedrich-Alexander-University Erlangen-Nuremberg, Kochstraße 6, 91054 Erlangen, Germany; 2 Philosophy and Ethics Group, Department of Industrial Engineering & Innovation Sciences, TU Eindhoven, De Zaale, Atlas 9.328, Eindhoven, the Netherlands

**Keywords:** data ethics, artificial intelligence, datafication, visibility, capability approach, oppression, vulnerability, injustice, recognition, bias, fairness

## Abstract

Datafication shapes and gradually transforms societies. Given this impact, issues of justice around data-driven practices have received more and more attention in recent years as shown, for example, by various reports and guidelines on artificial intelligence and data ethics. In this article, we elaborate on and defend two claims. First, these discourses on justice tend to center primarily around conceptions of fairness. We argue that justice in connection with datafication relates to, but ultimately encompasses more than, solely fairness. Second, although it is an important project to clarify what justice in connection with datafication encompasses, we argue that attention toward attitudes and practices of *data solidarity* have so far been largely overlooked. They are, however, indispensable as a catalytic element to advance toward data justice in practice.

## The bigger picture

New technologies such as big data, machine learning, and artificial intelligence are rapidly generating new opportunities and challenges for various social practices. At the same time, they raise important ethical questions. For example, for whom will these opportunities and advantages result in benefits, and who bears the burdens? A fundamental issue is which individuals and groups with their respective claims and particularities are involved and affected by the use of ever-increasing amounts of data and the inferences drawn from them. To navigate this terrain, reference is often made to the notion of justice. Such reference can be helpful to the extent that we are clear about what justice entails and what it takes to get there. In this article, we argue that justice requires us to make visible the claims of those left out, excluded, or disadvantaged in and around the use of data. To this end, we put forward the concept of data solidarity and examine its interplay with data justice.

## Teaser

Justice in and around data and artificial intelligence is often reduced to fairness. We follow proponents of the notion of data justice in arguing that the focus needs to be widened. We propose that in addition, a commitment to data solidarity is needed in which the concerns and interests of those who are currently insufficiently considered in social endeavors mediated by data are put to the forefront. Data solidarity is an important catalytic element to advance toward data justice.

## Introduction

We can hardly imagine a world without data. Data form the basis of a large variety of attempts to address urgent social problems and challenges. We use data and digital technologies to better understand various phenomena; to make predictions about medical, social, or even political processes; and to guide response efforts to public health crises, such as pandemics and epidemic outbreaks.

Data are produced, collected, processed, and used by different actors. Throughout all these processes, not only the amount of available data and the state of knowledge change, but also the possibilities and conditions of social actions. This interplay between the production and processing of data and the social transformation has prompted multidisciplinary discourses on datafication.^1^^,^^2^ These debates recognize that the processing of data^3^ from across all areas of our lives can fundamentally affect and shape social relations and the way data are generated, collected, and analyzed.

Given such impact on social relations and institutions, it is hardly surprising that issues of justice in connection with data are receiving more and more attention.^4^ Numerous reports and guidelines have appeared in recent years that attempt to address the question of what justice in dealing with data could mean. We concur that these documents provide important considerations around components of justice against the background of intensifying datafication. However, we are convinced that they also leave other aspects unarticulated and slide over important differences within the spectrum of accounts of justice. As we will argue, there is a tendency to equate and thus to conflate justice with fairness. This threatens to neglect that justice might encompass more and that any ideal concept of justice encounters already existing social structures of recognition and concrete experiences of discrimination and injustice. Highlighting and drawing on the work of Linnet Taylor, we spell out what else besides fairness might be needed to pursue data justice. We suggest that even a complete notion of data justice is in need of an additional element to become practical and ameliorate datafication and data-driven practices: data solidarity, understood as the commitment to remedy data-facilitated experiences of injustice.

## Fairness and beyond: Broadening the quest for justice in artificial intelligence and data ethics

Almost all of the numerous guidelines and frameworks on artificial intelligence (AI) ethics mention justice.^5^^,^^6^ However, the details vary. In their review entitled “The Global Landscape of AI Ethics Guidelines,”^5^ Jobin et al. write: “Justice is mainly expressed in terms of fairness, and of prevention, monitoring or mitigation of unwanted bias and discrimination. […] Whereas some sources focus on justice as respect for diversity, inclusion, and equality, others call for a possibility to appeal or challenge decisions, or the right to redress and remedy. Sources also emphasize the importance of fair access to AI, data, and the benefits of AI.”^5^

Such pluralism is understandable, given the broad, foundational scope of justice as “the first virtue of social institutions.”^7^ As such, it plausibly involves a multitude of different aspects and measures. It could therefore be a reason for caution that, as noted by Jobin et al.,^5^ there is a tendency to use justice and fairness interchangeably in ethics guidelines on AI. As one example, the EU High-Level Expert Group (AI HLEG) presents four guiding principles for the development and deployment of AI (human autonomy, prevention of harm, fairness, and explicability). There is obvious and acknowledged^8^ overlap with the four classic bioethics principles.^9^ Interestingly, however, there are also differences: the principle of beneficence is omitted; with explicability, a new principle is added; and instead of justice, the AI HLEG speaks of fairness. It is certainly debatable whether such a change of principles is necessary or desirable. But of particular interest for the focus of this article is the idea of replacing justice with fairness. Fairness is understood as encompassing “both a substantive and a procedural dimension. The substantive dimension implies a commitment to: ensuring equal and just distribution of both benefits and costs, and ensuring that individuals and groups are free from unfair bias, discrimination, and stigmatisation. […] The procedural dimension of fairness entails the ability to contest and seek effective redress against decisions made by AI systems and by the humans operating them.”^10^ Similarly highlighting the importance of distributive aspects, the European Group on Ethics demands that “AI should contribute to global justice and equal access to the benefits and advantages that AI, robotics and ‘autonomous’ systems can bring. […] We need a concerted global effort towards equal access to ‘autonomous’ technologies and fair distribution of benefits and equal opportunities across and within societies.”^11^

One prominent precursor of drawing a close connection between justice and fairness is Rawls, though three caveats are in order: first, Rawls himself actually assumes a conceptual difference between justice and fairness^12^ against the backdrop of which a proposed account of the former in terms of the latter is informative. Second, he unfolds one particular conception of what justice as fairness requires.^7^ Others who refer to justice, fairness, and/or their interplay dispute this conception^13^ and advance their own. Third, especially in his later works, Rawls countenances a kind of pluralism of justice when stating that “the content of public reason is given by a family of political conceptions of justice, and not by a single one. There are many liberalisms and related views, and therefore many forms of public reason specified by a family of reasonable political conceptions. Of these, justice as fairness, whatever its merits, is but one.“^14^ On these grounds, attention to Rawls should actually caution against tacit and unexplained equivocations between justice and fairness.

Precisely because of the many dimensions of justice if conceived of as the primary virtue of social institutions, there is a risk of eliding important aspects if narrow or overly reductive accounts of justice are put forward. There is no doubt that the dimensions of fairness mentioned by the AI HLEG (which is only one instance of the tendency captured by Jobin et al. and others) are important components of justice. Moreover, reflections on what fairness means in the context of our datafied lives mediated by algorithmic tools and how fairness could be embedded into technology are important, ongoing, and normatively rich tasks. Yet, when thinking about data justice, which we could construe as the first virtue of social institutions in a datafied and data-driven society, we should appreciate the significance of fairness without crowding out any further dimension of justice that might be overlooked if one is equated with the other. It is not misguided, yet possibly incomplete,^15^ to construe the quest for justice as involving reflection on whether a practice or outcome is fair.

To give only a few examples of what else deserves attention, consider how some maintain that justice is at least partly a matter of recognition^16^ among subjects, i.e., some form of respect, appreciation, or esteem for traits and actions of the recipient. Justice has material and distributive aspects, but involves recognition as either one essential dimension^17^ or its foundation.^18^ For example, Honneth reflects on what it would take to realize social justice vis-à-vis a minority community and suggests that recognition in the form of esteem is indispensable: “With the demand that a minority communal culture be socially esteemed for its own sake, the normative horizon of both the equality principle and the achievement principle is definitely exceeded. For it is no longer a matter of either ensuring, with the greatest possible value-neutrality, the equal opportunity of all subjects to realize their life goals, nor of as fairly as possible esteeming particular contributions to society as ‘achievements’, but rather the far more sweeping goal of respecting the cultural practices of a minority as something socially valuable in itself—as a social good.”^18^ According to this view, there are important distributive aspects to justice, but any normative commitment on (re-)distribution rests on relations of social recognition. This framework has already been applied to datafication, e.g., to spell out claims for data ownership.^19^

As another example, some authors emphasize that justice and injustice, if understood properly, are entangled with systemic and structural preconditions. Following a critique of purely distributive paradigms of justice, Young's seminal work highlights that “[t]he concepts of domination and oppression, rather than the concept of distribution, should be the starting point for a conception of social justice.”^20^ Domination amounts to “structural or systemic phenomena which exclude people from participating in determining their actions or the conditions of their actions.”^20^ She characterizes oppression as having the “five faces”^20^ of exploitation, marginalization, powerlessness, cultural imperialism, and violence. Importantly, these impediments to justice “involve matters which cannot easily be assimilated to the logic of distribution: decision-making procedures, division of labor, and culture.”^20^ The related worry echoing in debates on data ethics is that a focus on fairness will not be sufficiently sensitive to oppression. As Bui and Noble put it: “current and dominant frames of fairness within […] ‘ethical’ AI interventions often fail to consider and integrate notions and issues of structural and systemic inequality and power within their imaginaries and conceptualizations of the moral and ethical dimensions of AI and AI systems.”^21^ And as Kalluri highlights, organizations respond to potential harms arising from AI systems “with pledges to design ‘fair’ and ‘transparent’ systems, but fair and transparent according to whom? These systems sometimes mitigate harm, but are controlled by powerful institutions with their own agendas. At best, they are unreliable; at worst, they masquerade as ‘ethics-washing’ technologies that still perpetuate inequity. […] What is needed is a field that exposes and critiques systems that concentrate power, while co-creating new systems with impacted communities: AI by and for the people.”^22^

Finally, a mere focus on fairness could leave the significance of responsive action unarticulated. Justice is inherently tied to emancipatory movement and the active overcoming of oppression, whether experienced firsthand or observed occuring to someone else, and to break up structures that weave constraints against some into the social fabric. Likewise, it will not be enough on this understanding of justice to pursue the non-exacerbation of unfairness, as we appear to do when cautioning against, and trying to avoid, the perpetuation of bias by algorithmic tools.^23^ Besides mere non-exacerbation, action and policy facilitating emancipation and amelioration are right at the center of pursuits of justice.

Let us emphasize that this is not the place to endorse any of these accounts. Our goal is only to sketch them as options in argumentative space and to suggest that equating justice with fairness blurs these potentially important facets. This is not to say that uses of fairness will never implicate those things, which are all potentially within the scope of particular calls for fairness. However, fairness as such does not necessarily bring them to the forefront, and thus subsuming them all under the notion of fairness is imprecise at best. Neither is to say that these authors who elaborate on justice do not call for fairness, e.g., when highlighting the importance of fair conditions of opportunity,^24^ just that their accounts are not limited to fairness. More problematically, distributions and procedures can appear to satisfy conditions of fairness yet fail to recognize individuals and communities and/or be oppressive, unduly disciplining, etc., and hence unjust.

We suspend judgment on which causes factored into the observed tendency of AI ethics frameworks to focus on fairness and formalizations thereof. It is certainly possible that certain features of the subject matter itself contribute to this tendency. The utility of machine learning and AI partly flows from trying to translate social challenges into formalizable and operationalizable problems that can be solved, or at least partly addressed, through various forms of automation. It is then tempting to approach issues of fairness from within and by means of this very methodology. Intellectual investment into methods of machine learning might invite an orientation toward notions of fairness that are themselves formalizable and operationalizable and lend themselves to automated solutions—orientations that we might ultimately challenge and criticize.^25^, ^26^, ^27^ Again, we stay neutral on whether fairness and/or central aspects thereof necessarily resist formalization. Neither do we dispute that fairness is important or even essential to justice. Instead, the point is that even with the distributive, procedural, and substantive dimensions of fairness in place, we might worry that justice requires more than fairness in this sense.

In the recent past, more encompassing accounts have been put forward and have prompted commentators to speak of an “emergence of a new wave of ethical AI—one focused on the tough questions of power, equity, and justice that underpin emerging technologies, and directed at bringing about actionable change. It supersedes the two waves that came before it: the first wave, defined by principles and dominated by philosophers, and the second wave, led by computer scientists and geared toward technical fixes.”^28^ In the following, we focus on one specific, comprehensive theory of justice in connection with the digital: Linnet Taylor's account of data justice. One recurring theme in her work is that susceptibility to injustices mediated or facilitated by datafication is particularly high for populations that are already marginalized.^29^^,^^30^ In view of such dynamics, and taking up previous contributions on data justice,^31^, ^32^, ^33^ she proposes a framework that pursues human flourishing as the overarching aim of data justice and adopts a version of the *capability approach.*^34^, ^35^, ^36^ This approach conceives of capabilities as “the doings and beings that people can achieve if they so choose, such as being well-nourished, getting married, being educated, and traveling; functionings are capabilities that have been realized.”^37^ Unlike the accounts surveyed so far, Taylor explicates her call for data justice by reference to a demanding and explicit notion of “fairness in the way people are made visible, represented, and treated as a result of their production of digital data.”^29^

## Challenges for data justice

We have shown so far that fairness is an important aspect of justice, but that justice cannot simply be reduced to fairness. In the following, when we continue to speak of justice in dealing with data, we understand this to encompass two fundamental dimensions. They apply independently of the substantive theory of justice one endorses. First, data justice alludes to fundamental and universal standards of what is owed to individuals, in a sense to be specified by one's preferred substantive theory of data justice.^38^ Second, any ideal concept of data justice encounters already existing social structures of recognition and concrete experiences of discrimination and injustice.

To implement data justice and guarantee it for as many parts of society as possible, Taylor proposes three pillars: (in)visibility, (dis)engagement with technology, and non-discrimination.^29^ Each of them facilitates specific functionings of people vis-à-vis data. Upon systematic conversion, they give rise to or reinforce further capabilities, such as participation, access, and inclusion.

The first pillar, *(in)visibility*, comprises two levels: according to Taylor, this pillar of data justice demands that privacy claims are safeguarded and that necessary and appropriate forms of representation are guaranteed. In more concrete terms, a decisive contribution of data justice is to check again and again whether the claims and rights of all those who are affected by a possible use or non-use of certain data are also heard and respected.^39^ Against this background, Taylor, similar to scholars such as Abeba Birhane more recently,^40^ highlights that justice in dealing with datafied lifeworlds and automated decision making must essentially be measured by the extent to which those who are marginalized or made less visible by structural injustices are also rendered visible in their claims and rights. Understood in this way and resonating with our remarks above, justice in dealing with data should not be measured only by the extent to which already visible claims and rights are respected and addressed fairly in Taylor's sense.

Of course, it is not the case that visibility is necessarily bound to enhance justice. For example, McDonald shows by reference to the 2014 Ebola outbreak in West Africa how decisions on whether or not to make visible can themselves be precarious, and visibility at least potentially can be harmful, especially if decisions on whether, whom, and how to make visible need to be taken in emergencies such as epidemic outbreaks. He cautions that some are willing to endorse double standards for high-income (where privacy is privileged) versus low- and-middle-income settings (where people are faster to call for data sharing, in this case of mobile network data, that potentially undercuts individual rights).^41^

This points to the central challenge to assess what *equal* visibility looks like for different groups or entities. This is an issue especially if it is true that data justice depends on making visible again those who have either not been taken into account at all so far or whose claims and rights have become invisible owing to experienced injustices. As the philosopher Merleau-Ponty argues, visibility is not a feature that is possessed independently of the activity of agents, but one that must be continuously produced, maintained, and restored.^42^ These modes of making visible do not simply happen on their own, but often require overcoming resistance. It is precisely in places and situations in which structural elements of marginalization are at work that concrete practices and procedures of making visible are needed. In this process, it is important to define criteria and standards to maintain visibility. However, in order to make invisible and marginalized groups visible when dealing with data, such criteria and standards alone would not be sufficient. Rather, a clear focus on the concrete experiences of invisibility is needed to create visibility. Data justice, it could be pointed out, is dependent on conditions that it cannot achieve on its own. Someone must stand in the gap and be prepared to actively bring the claims of the invisible into the sphere of justice.

A second pillar, *(dis)engagement* with technology, involves autonomy in technology choices and the fair and appropriate sharing of benefits of datafication and data processing. Taylor emphasizes that this explicitly encompasses opportunities for data subjects to engage with, but also to withdraw from, data processing. This requires individual modes of control: “The freedom to control the terms of one's engagement with data markets is an essential component of any data justice framework because it underpins the power to understand and determine one's own visibility.”^29^

At the same time, however, there is a second central challenge in dealing with situations in which different claims to control do not coexist flawlessly but come into conflict. This issue should be all the more pressing for Taylor, as her normative guiding concept, human flourishing, while certainly a plausible guiding notion for data justice, is not without inherent tensions. Individual flourishing is dependent on social structures,^43^ and the flourishing of a community is related to the flourishing of its members as well as its coexistence with other communities. Yet, when tracing and seeking to strengthen human flourishing, we are likely to encounter situations in which the flourishing of a community will require at least partial constraints on the flourishing of particular individuals or other communities. Vice versa, some pursuits of individual flourishing appear reasonable in terms of promoting individual flourishing, yet end up jeopardizing or damaging communal flourishing. First, this illustrates that we must not only think about distributing benefits equitably, but also about ensuring that risks do not accrue disproportionately on the shoulders of only some. Second, all these interdependencies replicate in data governance, e.g., in cases where categorical prioritization of the privacy of some hampers socially beneficial data processing, or when expansive data processing that might deliver net benefits to a community or society constrains individual flourishing of some of its members. Analogous points apply to Taylor's focus on attending to the *needs* (rather than *rights*) of people: considering the fact that there will not always be harmony between individual needs, but also occasional competition between needs of some and those of others, plenty of questions about justice will remain on *how* the needs of different people ought to be satisfied.

The third pillar of *anti-discrimination* includes concrete possibilities to identify illegitimate bias and to have the power to prevent instances of discrimination. We might think, for example, of objecting to facial recognition algorithms that routinely misclassify non-white individuals or human resources (HR) recruiting algorithms and credit score assessments that disadvantage non-male applicants. Data justice requires concrete mechanisms for preventing these forms of discrimination for present and future uses and (further) developments of the systems.^29^

One central third challenge is how to establish clearly defined procedures to regulate how experiences of discrimination^15^ that have been uncovered can and must be dealt with.^44^ What is needed here are both transparently stated procedures and debates on the reasons and assumptions underlying the choice of these procedures. If it becomes apparent in the choice of databases to be included, in the programming of algorithmic systems, or in decisions related to the use of certain results of algorithmic systems that certain groups are underrepresented or not represented at all, data justice will depend crucially on the clear designation of mechanisms through which the integration of the respective groups can and should take place.

Such a constant integration will depend on the extent to which agents or institutions are defined and established that are responsible for implementing, critically evaluating, and ultimately enforcing these mechanisms. This is crucial not least because it does not suffice to simply name and jointly develop possible procedures to address injustices without considering the views of marginalized groups or individuals whose rights have been systematically disregarded. Responsibility and accountability for undue discrimination must be formalized, governed, and codified into socio-legal structures to bridge the gap between normative demands of data justice and concrete data practices.

As has become clear, Taylor's account is broader than the approaches that reduce justice to narrow conceptions of fairness; e.g., it addresses the three areas that the latter seemed to neglect: she considers oppression by devoting specific attention to phenomena of marginalization and inclusion in data-driven practices. She calls for responsive action when demanding the promotion of contextual “conversion factors” for translating between capabilities and functionings of data subjects. And while she does not explicitly deploy the notion of recognition, she does focus on fundamental human needs and prerequisites for human flourishing in her version of the capability approach. The noted challenges, rather than undermining her account, deserve ongoing consideration in its specification and implementation.

## Solidarity as a catalyst for data justice

These considerations on justice in dealing with data highlight the importance of at least three more specific questions. First, who is within the scope of data justice? Quite a few of the existing guidelines and reports appear to assume that this question has already been answered and that the answer is obvious: those whose claims and rights are visible. This is not wrong, but it overlooks a second important evaluative question: are all potential claims—including those that are perhaps currently invisible—considered, addressed, and included? Third, and picking up on Taylor's reflections: how can we succeed in including those who are left out by structural injustices in the negotiation of justice claims?

One promising approach to address all three questions could be to supplement the considerations on data justice with attention to *solidarity*. There are different understandings and definitions of solidarity.^45^, ^46^, ^47^ In the context of this discussion, we understand solidarity as shared practices of individuals or groups based on perceived similarities, in particular, in connection with their views and goals. These goals range from the intention to share risks and benefits, to create social bonds, or to recognize claims previously excluded from social practices.^48^^,^^49^ Thus, the point of solidarity can be best described as the creation and reinforcement of a social fabric^50^ through shared practices. As Tava has pointed out recently, there may be different occasions and motivations for solidarity, but a central one is the experience and/or observation of injustice.^51^ Whenever injustices arise and lead to certain individuals or groups being invisible or marginalized in their claims, there is a need for shared practices to reiterate their claims.^52^

Of course, it would be unwise to unconditionally romanticize solidarity and to neglect that enacting solidarity can in principle alleviate injustice, but also aggravate it or cause new kinds of injustice.^38^ As just one example, we might engage in the use of contact tracing applications in the context of a pandemic on the basis of solidarity with others in the community or society, some of whom might as a result of this very tracking activity become vulnerable to undue discrimination.^53^ These vulnerabilities might accrue inequitably across population segments, e.g., socioeconomic backgrounds, race, or ethnicity. For example, in demonstrations after the murder of George Floyd in 2020, there was—at least briefly—a concern that tracing data could be used by police officers to investigate protestors of the Black Lives Matter movement.^54^ Owing to the conceivability of cases such as these, we do not suggest that solidarity—in this case the willingness of individuals to share their data through tracing applications—is bound to promote data justice in a given context. Our point is simply that if we seek to promote justice, solidarity is an important catalytic element to get there.

## Toward data solidarity

Applying these fundamental ideas to the context of data justice, which, as Taylor reminds us, pertains to “the way people are made visible, represented and treated as a result of their production of digital data,”^29^ suggests that data justice too rests in important ways on solidarity. By extension to the terminology data justice, we propose to emphasize the need for *data solidarity.*

Data justice is dependent on shared practices of hearing the voices of others, in particular, those who remain neglected, and making their concerns our own.^55^ First, such shared practices create conditions on which data justice is built. It is arguably unrealistic to arrive at sustainable real-world arrangements of data justice without individuals engaging, at least at some point in the genesis of these arrangements, in shared practices of attending to and acting upon the concerns of others. Shared practices are needed to bring excluded and marginalized groups back into the realm of data justice and to make them and their concerns visible again. For example, enabling data subjects to be visible, and to become invisible if and when desired, is a target that will not be achieved merely by subscribing to it or by calling for it in the abstract. As Merleau-Ponty reminds us, visibility must be continuously *produced*.^42^ Visibility as a fundamental component of data justice requires continuous, concrete efforts to make those subjects visible who are currently invisible and to maintain and to foster the visibility of those who are about to fall outside the scope of visibility.

Nor should we stop at the level of visibility. Even if some populations are visible and represented in the evidence base, injustice can accrue further downstream when taking action: they might remain invisible, underrepresented, and overtly or structurally treated inadequately in decision-making processes and/or their outcomes. A case in point is the fact that in North America, coronavirus disease 2019 (COVID-19) has disproportionately affected Black communities as well as Indigenous communities such as Native Americans and Alaska Natives.^56^^,^^57^ As commentators point out, these disease burdens and the fact that they are coupled with and reinforced by decades of structural inequities and unfavorable social determinants of health are very visible in data, yet are not taken into consideration when distributing scarce medical resources. For example, vaccine allocation often proceeds without attending to socioeconomic aspects, and in view of such lack of attentiveness commentators call for considering such health inequities in the vaccine rollout.^58^^,^^59^ This is a case in point that even visible injustice well captured in data is unlikely to be alleviated unless solidarity-driven action is taken. These examples illustrate what we mean when suggesting that data solidarity has catalytic and ameliorative potential toward data justice.

Second, solidarity can play a role in detecting injustice. Often, individuals will become aware that they have been subjected to injustice only when they consider their experience in conjunction with the experiences of others. For example, being denied a credit loan, being subjected to law enforcement scrutiny, or being rejected in recruitment processes might seem innocuous in isolation, but will start to feel unjust especially once one realizes that others with whom one shares certain attributes consistently have similar experiences. Movements of solidarity that already address experiences of this kind within a society can be epistemically valuable in making these experiences salient *as* experiences of injustice. Likewise, observers might be alerted to potential injustices experienced by certain individuals or groups on the basis of observing the expressions and the formation of movements of solidarity.

Nevertheless, the assessment of whether an injustice has occurred, i.e., whether we ourselves have been subjected to an injustice or have observed (or become aware that we have overlooked) someone else being subjected to an injustice, can be a challenging epistemic task. While it is beyond the scope of our discussion to arrive at necessary and sufficient criteria for accomplishing this task, we do suggest that subjective experiences carry relevance by bringing potential occurrences of injustice to the forefront. Such reports can help in clarifying—to anyone seeking to enact data justice—who exactly is in need of solidarity and why. It allows those who make these reports to drive debates and elicit solidarity of others in a bottom-up fashion and to raise issues that might otherwise remain overlooked. In view of individuals' first-person knowledge about how they are treated, which opportunities they enjoy, and which obstacles they still face in social space, this bottom-up perspective is epistemically valuable for assessing whether or not societal arrangements are just.

When someone makes a report to this effect, this gives a *prima facie* reason to hear their concerns. In deeming subjective experiences of injustice relevant, we consider such experiences neither necessary nor sufficient for there actually being an injustice. Sometimes people might simply be unaware that they have been subjected to an injustice and thus will not report such a subjective feeling. Other times they might falsely perceive an injustice when, on reflection, no injustice has occurred (both possibilities are independent of our preferred substantive criteria for what makes something unjust). These already intricate questions become even more complicated in a “black box society,”^60^ in which data-driven and automated injustice can arise and prevail behind the backs of both the agents involved in bringing about the injustice and the agents affected by it.

Third, not just any kind of shared practices of attending to and acting upon the concerns of others promotes justice. Solidarity is not in and of itself directed toward justice, i.e., dedicating ourselves to the ends of certain individuals or groups does not automatically lead to arrangements that are just. One could just as easily identify with people who are already privileged or who oppose and prevent the consideration of those who are unduly neglected within and through datafied environments. In order for solidarity to unfold catalytic potential that is also ameliorative, it is essential that it submits to and is oriented toward standards of justice.

Ideally, such shared practices are translated and formally encoded into rules, standards, and structures that bind and robustly shape practice. Solidarity certainly can (and often does) have an affective dimension, for example, when we identify and empathize with those with whom we declare solidarity, potentially together with an appreciation and valuation of their struggle, integrity, or virtue. Formalization and codification of solidarity might well be preceded or triggered by affect, but importantly goes beyond it by ideally converting attitudes of solidarity into consistent action. This also applies to formalization and codification necessary to ensure accountability and redress for data injustices: data justice, including but not limited to the ability to challenge bias and to prevent discrimination, will be possible and sustainable only if there is accountability for data injustice, and data subjects are empowered to enforce their claims in the face of data injustices.

To produce and facilitate (in)visibility and other pillars of Taylor's notion of data justice, it is necessary to not only entertain the possibility of, but also to act upon, the conscious prioritization of those who have remained insufficiently considered up to now. Agendas must be set in ways that decidedly elevate their concerns and interests over others, especially in view of the fact that the goal of promoting human flourishing will often point in various different, mutually incompatible directions. In framing and shaping the engagement with technology, agents will encounter a multitude of sometimes mutually incompatible interests and concerns related to human flourishing and needs. In working toward data justice, they will be forced to negotiate and deliberate on *prioritizing* some concerns over others. These processes will always have to attend to context-specific needs, considerations, and institutional settings and arrangements. In this sense, considerations of data solidarity are unavoidable: in deliberating on tradeoffs in a datafied and data-mediated lifeworld, we cannot get around taking a stance on the question with whom solidarity is declared and enacted, i.e., whose concerns take precedence.

## Conclusions

Our discussion has identified two grades of incompleteness in debates on justice in connection with datafication. First, there is a tendency to reduce justice to overly narrow senses of fairness. We have argued that data justice requires more than focusing on fairness in data or data-processing algorithms. Taylor is right to widen the scope to the full spectrum of how data affect and interact with human flourishing. Second, even the most encompassing notions of justice in and around the digital are in need of further supplementation to effectively ameliorate data-intensive practices. Besides awareness of what data justice requires, in practice it will be vital to *catalyze change* to get there and to overcome challenges related to the pursuit of the ideal of data justice in the face of data injustices in a non-ideal world. We have proposed that data justice is intertwined with solidarity in the sense of making the concerns and interests of others one's own and to commit to including them into social endeavors mediated by data. Only with the readiness to engage and commit to taking up and elevating concerns of those who, in Taylor's terms, are so far left invisible, unwillingly visible, unfree to engage or disengage with datafication, and affected by bias but unable to challenge discrimination, can we get closer to realizing data justice. Besides clarity about the contours of data justice, there is a fundamental need for an ameliorative dynamic that starts with us, the agents of datafication who reflect on, debate, and/or shape data practices, technologies, innovation, and policy.

For a data governance that invokes justice as one of its central principles, the reflections on data solidarity proposed by this article call for giving the concrete experiences of marginalization and experiences of injustice in data-intensive contexts a conceptual place. Here, data solidarity requires us to continually check the procedures and arrangements of justice to ensure that all claims for recognition and respect have been heard and considered.
